# Phenacylation of 6-Methyl-Beta-Nitropyridin-2-Ones and Further Heterocyclization of Products

**DOI:** 10.3390/molecules25071682

**Published:** 2020-04-07

**Authors:** Eugene V. Babaev, Victor B. Rybakov

**Affiliations:** Chemistry department, Moscow State University, Leninskie gory, 1 str.3, Moscow 119899, Russia; rybakov20021@yandex.ru

**Keywords:** Phenacylation of beta-nitropyridin-2-ones, 8-nitro-5-RO-indolizines, oxazole-pyrrole ring transformation

## Abstract

Reaction between the derivatives of 6-methyl-beta-nitropyridin-2-one and phenacyl bromides was studied, and the yields observed were extremely low. The pyridones were converted via chloropyridines to methoxyderivatives, which were *N*-phenacylated. N-Phenacyl derivatives of 4,6-dimethyl-5-nitropyridin-2-one under the action of base gave 5-hydroxy-8-nitroindolizine and under acidic conditions gave 5-methyl-6-nitrooxazole[3,2-*a*]pyridinium salt, which underwent recycization with MeONa to 5-methoxy-8-nitroindolizine.

## 1. Introduction

**Scheme 1:**
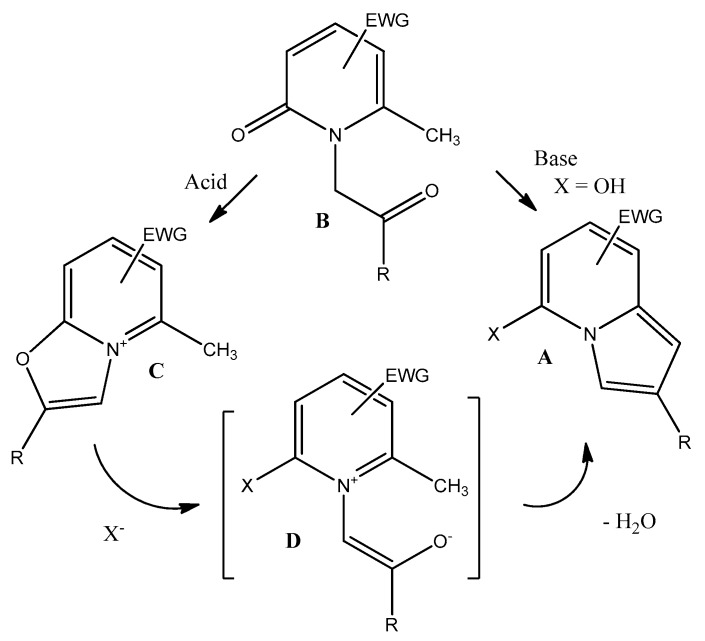
Possible transformations of *N*-(2-oxoethyl)-6-methylpyridin-2-ones.

Indolizine is an important member of the class of heterocyclic compounds, and many alkaloids have in their structures a saturated or aromatic indolizine moiety. While the chemistry of indolizines has been widely investigated [1], the chemistry of 5-substituted indolizines (**A**, Scheme 1) remains very poor because there are only a few reliable ways for their synthesis. Thus, there are examples of electrophilic substitution of indolizines lithiated at C-5 [2,3,4] and S_N_H reaction of 8-nitroindolizines at this position [5]. The standard Tchitchibabin reaction requires interaction of 6-substituted 2-picolines and alpha-bromoketones; by steric reasons, however, this reaction is almost impossible for 5-X-indolizines. Exceptional case is the cyclization of pyridones **B** (Scheme 1) bearing acceptor (EWG) group at beta-position [6,7]. The last methodology developed in our laboratory is the recyclization of 5-methyl substituted oxazolo[3,2-*a*]pyridinium salts **C** via pyridinium betaine **D** (Scheme 1), which leads to 5-substituted indolizines [8,9]. In turn, the salts **C** are available via acidic cyclization of pyridones **B** [10]:

The aim of this work was to test the validity of Scheme 1 for the case of more powerful acceptor a nitro group (EWG = NO_2_) by choosing the 6-methyl-beta-nitropyridin-2-ones as the parent compounds. Obtained by such strategy 6- or 8-nitroindolizines with the substituent X at position 5 may undergo easy nucleophilic substitution at C-5. In addition to the route from **B** to **A**, one could consider one more alternative strategy from **C** to **A**.

## 2. Results and Discussion

### 2.1. Synthesis of Isomers and Homologues of Beta-Nitro-6-Methylpyridin-2-One (**1a**–**d**)

**Scheme 2:**
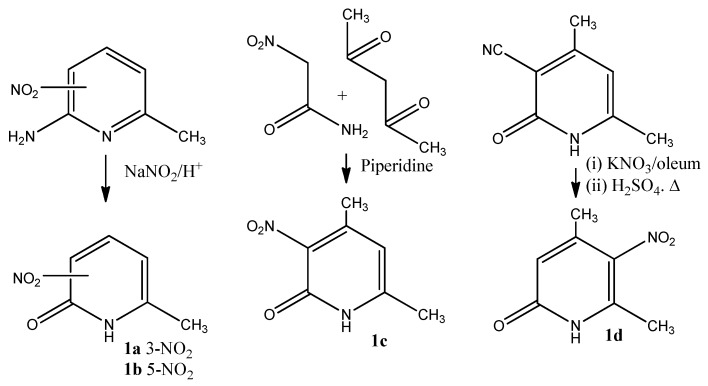
Synthesis of the parent homologues of beta-nitro-6-pyridin-2-ones.

The simplest scheme for the synthesis of 3- and 5-nitro derivatives of 6-methylpyridin-2-one was the diazotation of 2-amino-3(5)-nitro-6-picolines, which, in turn, are available by nitration of commercial 2-amino-6-picoline to 2-nitramino-6-picoline (95%) and its further acidic rearrangement (51%) and steam distillation (leading to 39% of 5-nitro- and 15% of 3-nitropyridin-2-ones). By this way (Scheme 2) both isomers, namely 6-methyl-3-nitropyridin-2-one (**1a**, yield 32% [11]) and 6-methyl-5-nitropyridin-2-one (**1b**, yield 25% [12]), were obtained; the low yield at the last step may be due to high solubility of products in acetone for recrystallization.

In addition, two 4-methyl homologues of pyridones **1a**,**b** were described in literature. To prepare 4,6-dimethyl-3-nitropyridin-2-one (**1c**) we performed the cyclization of acetylacetone with nitroacetamide and obtained the target compound **1c** with the yield 37% [13]. For preparation of nitroacetamide we used the isonitroacetoacetic ester, which was dangerous for synthetic chemists due to ability to detonate above 100 °C. (In addition, freshly isolated dry nitroacetamide was capable to self-ignition upon contact with air.) Finally, the synthesis of 4,6-dimethyl-5-nitropyridin-2-one (**1d**, yield 19% [14]) was achieved in two steps by nitration of Guaresci pyridine (obtained with the yield 93% and nitrated with the yield 47%) and consequent removal of the CN group in diluted sulfuric acid (low yield at this step may be due to solubility of product both in acid and in alkali used for neutralization).

### 2.2. Attempts of Direct Phenacylation of Homologues of Beta-Nitropyridin-2-Ones

Alkylation (and phenacylation) of pyridin-2-ones may occur either at *N*- or O-atoms [9]. Based on the literature data of reaction of phenacyl bromides with 4,6-dimethylpyridin-2-ones (bearing electron withdrawing group CN, CONH_2_, CO_2_R at C-5) [6,7,15,16,17,18]) we expected that alkylation of sodium salts of 6-methyl-beta-nitropyridin-2-ones (**1a**–**d**) would also proceed at the nitrogen atom. As an additional argument, Na-salt of 5-nitropyridin-2-one also underwent *N*-phenacylation [19]. We believed that the resulting mixtures of *N*- and O-phenacyl derivatives from **1a**–**d** could be chromatographically separated, since it is often mentioned in the literature that the chromatographic behavior of N- and O-isomers is different.

It turned out, however, that when trying to phenacylate the Na-salt of nitropyridones **1a**–**d**, the reactions proceeded with an extremely low yield. As can be seen, a combination of factors (a sterically hindered nitrogen atom deactivated by a nitro group) prevents N-alkylation. The result obtained completely excluded the opportunity to implement our planned strategy. In search of a possible solution to the problem, we drew attention to the other strategy described in the literature for the regioselective synthesis of pyridones with a phenacyl residue at the nitrogen atom using 2-methoxypyridines [9,20,21]. In this case, O-demethylation in the intermediate salt was apparently due to the attack of a rather weak nucleophile, the bromide ion. As a result, the methyl group acted as a protective group, and this method allowed selective and reliable preparation of *N*-phenacyl derivatives. For our purposes, we should use 2-methoxy-beta-nitropyridines (with a kind of protection—a methoxy group, which not only prevents competitive O-alkylation but also acts as a donor residue that promotes *N*-alkylation).

### 2.3. Synthesis of Beta-Nitro-2-Methoxypyridine Homologues and Their Phenacylation

**Scheme 3:**
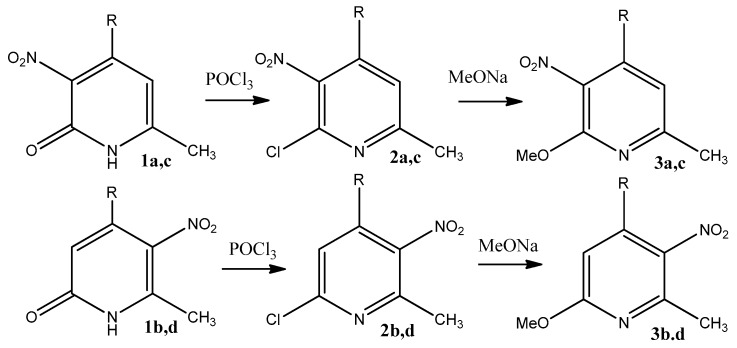
Conversion of beta-nitropyridin-2-ones to 2-cloro- and 2-methoxy derivatives. **1**–**3**: **a**, **b** R = H; **c**, **d** R = CH_3_.

An analysis of the literature showed that it is not a serious problem to convert the beta-nitropyridin-2-ones into 2-methoxypyridines with the intermediacy of 2-chloropyridines. The conversion of pyridin-2-ones to 2-chloropyridines proceeded with high yields upon boiling with POCl_3_ (Scheme 3), and the melting points of the obtained chloropyridines **2a**–**d** coincided with the published data (Table 1). The next stage—the replacement of Cl with a MeO group—was carried out by boiling with MeONa in methanol. The yields and melting points of the obtained substances **3a**–**d** are shown in Table 1.

**Scheme 4:**
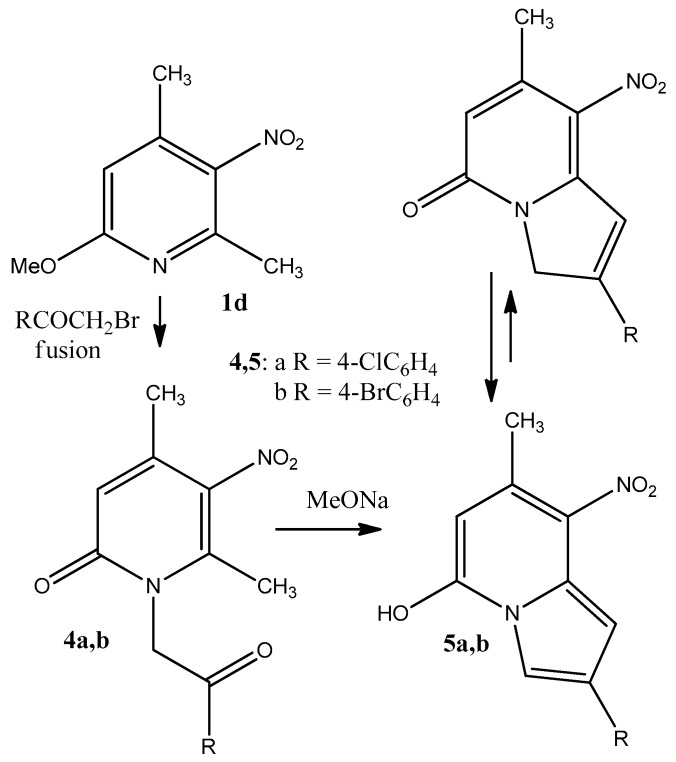
Phenacylation of nitropyridones and consequent cyclization.

**Figure 1:**
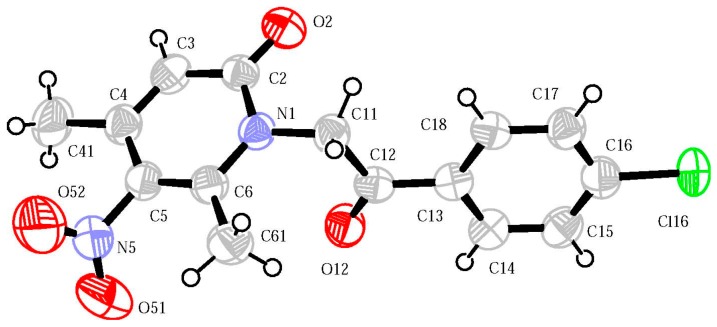
The general view of molecular structure of **4a** in representation of atoms by displacement parameters ellipsoids (p = 30%).

After numerous attempts to phenacylate 2-methoxypyridines **3**, we found the only acceptable method—melting of the starting reagents, Scheme 4. Such melting of reagents (in comparison with their boiling in acetonitrile) increased the yields 17 times, and they achieved 35%. The structure of *N*-phenacyl derivatives clearly followed from the spectral data. The ^1^H-NMR spectra of phenacylpyridones **4a**,**b** contain the expected signals of the phenacyl residue and a fragment of nitropyridone. The final confirmation of the structure of the *N*-phenacylpyridone **4a** was obtained by X-ray diffraction, Figure 1. The yields for other methoxypyridines (**3a**–**c**) were much lower.

### 2.4. Cyclocondensation of N-Phenacylpyridones under Basic Condition

For studies of the cyclization of phenacylpyridones, we used compounds **4a**,**b** obtained in acceptable quantities (see Experimental section). In the solution of **4a**,**b** in MeOH, an intense dark red color is observed. The neutralization of the reaction mixture allowed us to identify individual powdery compounds of dark red color. Their solutions turned black-green. The data of ^1^H- and ^13^C-NMR spectra showed that both compounds are substituted indolizines **5a**,**b** with the structure of 5-hydroxy tautomers, Scheme 4. This observation contradicted to the structure of 6-CN-5-hydroxy indolizines to which 5-oxo-3-CH_2_-type of tautomers was assigned [6,7]. In the ^1^H-NMR spectrum, we observed three singlets (in addition to multiplet of aryl residue and CH_3_ singlet). Two of them were from protons H-1 and H-3 of the pyrrole fragment, and the third one—at 4.4–5.4 ppm—was a singlet of the proton H-6 of the pyridine fragment. The last peak was shifted to a high field due to the ortho-located hydroxy group, whose signal appeared as a broadened peak in the region of 3.1–3.4 ppm. The confirmation that in this case indolizine existed precisely in the hydroxy form was the appearance in the IR spectrum of the characteristic vibration frequency of the OH group at 3437 cm^−1^.

The reasons why 5-hydroxy (oxo) indolizines existed in the oxy or oxo form depended, obviously, on the nature of the additional acceptor substituent in the pyridine nucleus. In the case of 6-cyano derivatives, the tautomeric equilibrium was completely shifted towards the oxo form, while in the case of 8-nitroindolizins, the oxy form prevailed. These groups were likely to have different effects on the acidity of the 5-OH group and the basicity of the C-3 atom of the pyrrole moiety (onto which the proton of the hydroxyl group can migrate).

By analogy with 6-cyanoindolizinones [7], we expected that the action of phosphorus oxychloride on the corresponding nitroindolizinoles would lead to the replacement of 5-oxo/oxy-group with chlorine. It turned out, however, that when both hydroxyindolizines were heated with POCl_3_, complete resinification was observed. Probably the phenolic nature of the obtained indolizines somehow prevented the occurrence of such a transformation.

### 2.5. Synthesis of 5-Substituted Indolizine via Oxazolopyridinium Salt

As we have seen, it was possible to obtain intermediate compounds, representatives of a previously unknown class of 5-hydroxyindolizines, and their structure was very interesting. It turned out, however, that these compounds were unpromising for introduction of any other functions to position 5 (since they cannot be converted into 5-chloro derivatives). This can mean that it was completely impossible to vary the residue at position 5 in the series of 6(8)-nitroindolizines within the framework of our chosen strategy.

**Scheme 5:**
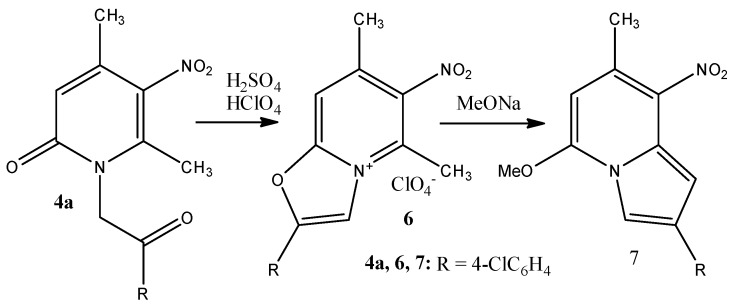
Closure of oxazolium ring in N-phenacylpyridone and further recyclization.

**Figure 2:**
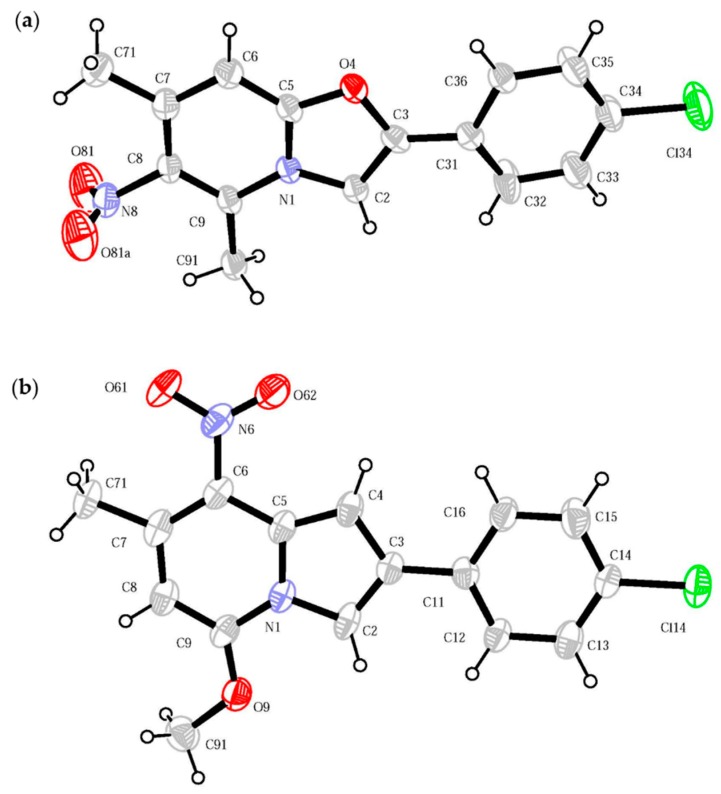
The general view of molecular structures of **6** (**a**) and **7** (**b**) in representation of atoms by displacement parameters ellipsoids (p = 30%). A perchlorate anion for **6** and solvate acetone molecule for **7** are omitted for clarity.

We were able to show that the precursors of 5-hydroxyindolizines, phenacylpyridones **4**, were very promising synthons for producing 6 (8)-nitroindolizines with a substituent other than the hydroxy group in position 5. It is known that such phenacylpyridones can close two different cycles under the action of bases and acids. In the last case cyclodehydration may lead to closure of the oxazolium cycle. We found that, under the action of concentrated sulfuric acid, phenacylpyridone **4a** were smoothly cyclized to the corresponding oxazolopyridinium cation **6**, isolated in the form of perchlorate, Scheme 5. In the ^1^H-NMR spectrum of the obtained salt, a singlet in low field appeared at 9.72 ppm, which corresponded to the proton of the newly formed oxazolium ring. The final proof of the structure of the obtained heterocycle **6** was obtained by X-ray diffraction, Figure 2a.

The resulting compound **6** was capable of easily reacting with nucleophiles. It turned out that in the reaction with MeONa, compound **6** underwent a rearrangement with the destruction of the oxazolium ring and the closure of a new pyrrole ring, forming 5-methoxy-8-nitroindolizine **7**, Scheme 5. The structure of the obtained compound **7** was unambiguously proved by the X-ray diffraction method, Figure 2b. (In the lattice there is a solvent molecule—acetone.)

Interestingly, the obtained 5-methoxy-8-nitroindolizine **7** was isostructural to the 5-hydroxy-8-nitroindolizines **5a**,**b** obtained above. Comparing the electronic absorption spectra of these two isostructural (and pi-isoelectronic) systems, one can conclude that the structure of the bands was similar.

## 3. Materials and Methods

### 3.1. General Information

^1^H-NMR spectra were recorded on a Bruker AC 400 instrument (Bruker, Bremen, Germany, operating frequency 360 MHz,), ^13^C-NMR spectra were recorded on a frequency of 100 MHz. Chemical shifts are measured on a δ-scale and are given in parts per million; *J* given in Hertz. The reaction progress was monitored by TLC on Silufol UV-254 plates (Merck KGaA, Darmstadt, Germany), and the TLC manifestation was carried out by UV radiation (wavelengths of 254 and 365 nm), iodine vapor, oxidation in a sulfuric aqueous solution of KMnO_4_, Ehrlich reagent or ninhydrin. Chromatographic separation was performed on columns or glass plates using silica gel with a particle size of 40–60 μm (Merck, KGaA, Darmstadt, Germany). Reagents from Acros (Fisher Scientific, Leicestershire, UK), Merck (Merck KGaA, Darmstadt, Germany) and Aldrich (Sigma-Aldrich Company Ltd., Dorset, UK) were used as starting materials for the syntheses. They were introduced into the reactions without additional purification. Elementary analysis data for new compounds are given in Table 2.

### 3.2. Synthesis

6-Methyl-3- and 5-nitropyridin-2-ones (**1a**,**b**) were obtained by diazotation of 2-amino-3(5)-nitro-6-picolines [11,12]. 4,6-Dimethyl-3-nitropyridin-2-one (**1c**) obtained by cyclization of acetylacetone with nitroacetamide [13]. 4,6-Dimethyl-5-nitropyridin-2-one (**1d**) was obtained in two steps by nitration of Guaresci pyridine and further hydrolysis [14].

Studying of phenacylation of sodium salt 4,6-dimethyl-5-nitropyridin-2-one (**1d**). In methanol. To a solution of 0.483 g (0.021 mol) of sodium metal in 100 mL of absolute methanol, 3.36 g (0.02 mol) of nitropyridone **1d** and 4.9 g (0.021 mol) of p-chlorophenacyl bromide are added with vigorous stirring. The mixture was stirred for 12 h at 55–60 °C and controlled by TLC. Only traces of product could be observed. In benzene/xylene. To a benzene suspension of 0.5 g (2.9 mmol) sodium salt of pyridone **1d** 0.8 g (35 mmol) of p-bromophenacyl bromide was added. After 60 h at room temperature TLC monitoring showed the presence of only starting materials. The solvent was evaporated and the mixture was dissolved in o-xylene and boiled for another 6 h. TLC control showed the presence of only traces of products.

Conversion of pyridin-2-ones (**1a**–**d**) to 2-chloropyridines (**2a**–**d**). The mixture of 0.036 mol of nitropyridone-2 (**1a**–**d**), 4 mL of POCl_3_ and 0.036 mol of PCl_5_ was maintained at 120 °C for 4 h. The mixture was left at 120 °C in a Wood alloy for 1.5 h. The mixture was cooled to RT, poured into excess of ice water and the brown precipitate was filtered off, dried and recrystallized from hexane. The yields, m.p. of products **2a**–**d** and literature references are given in Table 2.

Conversion of 2-chloropyridines (**2a**–**d**) to 2-methoxypyridines (**3a**–**d**). To a solution of MeONa (obtained by dissolving 0.388 g (0.017 mol) of sodium metal in 15 mL of absolute MeOH) 0.016 mol 2-chloropyridine **2a**–**d** was added. The mixture was boiled for 4 h, the precipitated NaCl was filtered off, the filtrate was evaporated and the residue was chromatographed on a column (SiO_2_, chloroform). The yields, m.p. of products **3a**–**d** and literature references are given in Table 1.

*N*-(p-chlorophenacyl)-4,6-dimethyl-5-nitropyridin-2-one (**4a**). A mixture of 1.3 g (0.007 mol) of methoxypyridine **3e** and 1.75 g (0.007 mol) of p-chlorophenacyl bromide was dissolved in 10 mL of acetonitrile and the solution was boiled for 30 h. The solvent was evaporated in vacuo. The resulting mixture was kept for 17 h at 100 °C and 15 h at 120 °C, cooled to room temperature and chromatographed on a column (SiO_2_, chloroform). Product **4a** (0.14 g) was obtained. Yield 6%; m.p. 190–191 °C. ^1^H-NMR Spectrum (DMSO-*d_6_*): 8.10 (2H, m, Ar); 7.57 (2H, m, Ar); 6.30 (1H, s, 3-H); 5.65 (2H, s, CH_2_); 2.30 (3H, s, 6-CH_3_); 2.22 (3H, s, 4-CH_3_). The molecular structure is shown in Figure 1 [28].

*N*-(p-bromophenacyl)-4,6-dimethyl-5-nitropyridin-2-one (**4b**). Obtained in a similar manner from methoxypyridine **3d** and p-bromophenacyl bromide. Yield 8%, m.p. 192 °C. ^1^H-NMR Spectrum (CDCl_3_): 7.92 (2H, m, Ar); 7.78 (2H, m, Ar); 6.94 (1H, s, 3-H); 5.74 (2H, s, CH_2_); 2.30 (3H, s, 6(4)-CH_3_); 2.25 (3H, s, 4(6)-CH_3_).

Phenacylation of methoxypyridine **3d**: synthesis optimization without use of a solvent.

(1) The reaction of **3d** with p-chlorophenacyl bromides was carried out in a sealed glass ampoule, the reaction mass was heated in an oven for 25 h at temperature of 120–150 °C. TLC analysis in pure chloroform showed the presence of the desired products. After chromatography, 0.378 g of a pure yellow substance, identical in TLC and m.p. with the N-isomer **4a**. Yield ~10%.

(2) The substances were placed in a 50 mL flask equipped with a reflux condenser. The mixture was kept at 120 °C for 7 h in a Wood alloy. After 5 h of boiling, the yellow liquid began to accumulate on the walls of the flask and flowed down. TLC analysis showed that it is phenacyl bromide. Then, after the cessation of gas formation, the temperature was raised to 200 °C (continued gas formation), and the mixture was kept at this temperature for another 3 h. The reaction mixture was applied to silica gel and chromatographed with CHCl_3_, then CHCl_3_—EtOH. Recrystallized from acetone. The yield of N-isomer **4a** was 15%.

(3) 4 g of methoxypyridine **3d** and a twofold excess of phenacyl bromide were taken. The substances are placed in a 50 mL flask equipped with a reflux condenser. The mixture was heated in a Wood alloy at 125 °C for 20 h. After this, the mixture was poured into a large amount of boiling petroleum ether and the precipitate was filtered off. A petroleum extract containing pure starting materials was reacted back. The precipitate was purified from the crude oil by chromatography, eluting with chloroform. The yield of the target product **4a** was 35%.

Synthesis of 5-hydroxy-8-nitroindolizines **5a**,**b** (General methodology). A total of 0.6 g of phenacylpyridone **4a** (1.6 mmol) was dissolved in 100 mL of MeOH. The calculated amount of Na (38 mg) was dissolved in 50 mL of MeOH. A solution of MeONa was added with stirring to a solution of phenacylpyridone. The solution turned raspberry colored. In 30 min after the start of the reaction the calculated amount of HOAc was added to neutralize MeONa. The reaction mixture was diluted with diethyl ether, and the product and NaOAc precipitated. The solvent was distilled off on a rotary evaporator, the mixture was dissolved in a minimum amount of acetone and filtered from NaOAc. The solution was evaporated giving a brick-red substance, 2-p-chlorophenyl-5-hydroxy-7-methyl-8-nitroindolizine (**5a**). Yield 92%. m.p. > 250 °C (decomp.). ^1^H-NMR Spectrum (CD_3_OD): 7.80 (1H, m, 3-H); 7.72 (2H, m, Ar); 7.47 (2H, m, Ar); 7.29 (1H, m, 1-H); 5.37 (1H, s, 6-H); 3.40 (1H, br s, OH); 2.57 (3H, s, CH_3_).

2-p-Bromophenyl-7-methyl-8-nitro-5-hydroxyindolizine (**5b**). Yield 90%, m.p. > 250 °C (decomp.). ^1^H-NMR Spectrum (CDCl_3_): 7.34 (1H, m, 3-H); 7.17 (2H, m, Ar); 7.08 (2H, m, Ar); 6.82 (1H, m, 1-H); 4.37 (1H, s, 6-H); 3.10 (1H, br s, OH); 2.13 (3H, s, CH_3_).

2-(p-Chlorophenyl)-5,7-dimethyl-6-nitrooxazolo [3,2-*a*]pyridinium perchlorate (**6**). A mixture of 0.1 g (0.3 mmol) of phenacylpyridone **5a** and 1 mL of concentrated sulfuric acid was maintained at 22 °C for 18 h. Then, 0.2 mL of 70% HCiO_4_ was added to the mixture, incubated for 1 h, poured into 100 mL of absolute ether and the precipitate formed was filtered off. The yield of perchlorate is 92%; m.p. 295–297 °C (decomp.). ^1^H-NMR Spectrum (DMSO-*d_6_*): 9.72 (1H, s, 3-H); 8.60 (1H, s, 8-H); 8.07 (2H, m, Ar); 7.82 (2H, m, Ar); 2.89 (3H, s, 5-CH_3_); 2.67 (3H, s, 7-CH_3_). ^13^C-NMR (DMSO-*d_6_*): 15.4; 18.5; 110.6; 112.1; 122.6; 127.5; 130.1; 137.1; 137.8; 144.8; 145.8; 152.2; 152.4. X-Ray data see Figure 2a. The molecular structure is seen in Figure 2a (perchlorate anion is omitted for clarity) [29].

2-(p-Chlorophenyl)-5-methoxy-7-methyl-8-nitroindolizine (**7**). A total of 200 mg (0.49 mmol) of oxazolopyridinium salt **6** was added to a solution of 10 mg of sodium in 10 mL of methanol. The mixture was kept for 1 day at 22 °C and the precipitate formed was filtered off. Product **7** (110 mg) was obtained. Yield 73%, m.p. 175–176 °C. ^1^H-NMR Spectrum: 7.50 (6H, m, Ar); 5.73 (1H, s, 6-H); 4.22 (3H, s, O-CH_3_); 2.69 (3H, s, CH_3_). The molecular structure see Figure 2b (solvate acetone molecule is omitted for clarity) [30].

### 3.3. X-ray Diffraction Studies

For single crystals of compounds **4a** and **6**, the experimental intensities of diffraction reflections were obtained on a CAD-4 diffractometer (λ Cu Kα radiation, graphite monochromator, ω scanning at room temperature, Enraf-Nonius, Delft, Netherlands), for compound **7** on a CAD-4 diffractometer (λ Mo Kα radiation, graphite monochromator, ω-scan, at room temperature). All subsequent calculations were performed as part of the SHELX software package [31]. The crystallographic data for the studied structures were deposited in the Cambridge Structural Database with the numbers CCDC 1986879 for **4a**, CCDC 1986629 for **6**, CCDC 1984804 for **7**.

## 4. Conclusions

In conclusion, the reaction between the derivatives of 6-methyl-beta-nitropyridin-2-one (**1a**–**d**) and phenacyl bromides did not occur for steric and electronic reasons. However, 4,6-dimethyl-2-methoxy-5-nitropyridine **3d** on fusion with bromoketones could be *N*-phenacylated. Its *N*-phenacyl derivatives **4a**,**b** under the action of base gave 5-hydroxy-8-nitroindolizines (**5a**,**b**) and under acidic conditions gave 5-methyl-6-nitrooxazole[3,2-*a*]pyridinium salt **6**, which underwent recycization with MeONa to 5-methoxy-8-nitroindolizine **7**.

## Figures and Tables

**Table 1:** Yields, mp and literature references for compounds **2a**–**d** and **3a**–**d**.

No	Yield, %	Mp	Mp, Lit	Reference
**2a**	45	68	70–70.8 67–69	[22] [23]
**3a**	32	57	57–58	[24]
**2b**	25	54–55	54	[25]
**3b**	36	64	64–65	[26]
**2c**	64	47	47–48	[27]
**3c**	90	104–105	104	[28]
**2d**	85	54	54–55	[27]
**3d**	92	59–60	60	[14]

**Table 2:** Elementary analysis data for all new compounds.

Compound (No.)	Formula	Calculated			Found		
		C	H	N	C	H	N
*N*-(p-chlorophenacyl)-4,6-dimethyl-5-nitropyridin-2-one (**4a**)	C_15_H_13_ClN_2_O_4_	56.17	4.09	8.73	55.82	4.13	8.68
*N*-(p-bromophenacyl)-4,6-dimethyl-5-nitropyridin-2-one (**4b**)	C_15_H_13_BrN_2_O_4_	49.34	3.59	7.67	49.13	3.62	7.64
2-p-chlorophenyl-5-hydroxy-7-methyl-8-nitroindolizine (**5a**)	C_15_H_11_ClN_2_O_3_	59.52	3.66	9.25	59.13	3.71	9.19
2-p-Bromophenyl-7-methyl-8-nitro-5-hydroxyindolizine (**5b**)	C_15_H_11_BrN_2_O_3_	51.90	3.19	8.07	51.51	3.25	8.01
2-(p-Chlorophenyl)-5,7-dimethyl-6-nitrooxazolo [3,2-*a*]pyridinium perchlorate (**6**)	C_15_H_12_ClN_2_O_3_*ClO_4_	44.69	3.00	6.95	44.40	3.05	6.90
2-(p-Chlorophenyl)-5-methoxy-7-methyl-8-nitroindolizine (**7**)	C_16_H_13_ClN_2_O_3_	60.67	4.14	8.84	60.29	4.18	8.79

* means ionic compound.
